# Strategies for improving production performance of probiotic *Pediococcus acidilactici* viable cell by overcoming lactic acid inhibition

**DOI:** 10.1186/s13568-017-0519-6

**Published:** 2017-11-27

**Authors:** Majdiah Othman, Arbakariya B. Ariff, Helmi Wasoh, Mohd Rizal Kapri, Murni Halim

**Affiliations:** 1 Department of Bioprocess Technology, Faculty of Biotechnology and Biomolecular Sciences, Universiti Putra Malaysia, 43400 Serdang, Selangor Malaysia; 2 Bioprocessing and Biomanufacturing Research Center, Faculty of Biotechnology and Biomolecular Sciences, Universiti Putra Malaysia, 43400 Serdang, Selangor Malaysia

**Keywords:** Lactic acid bacteria, Lactic acid, Extractive fermentation, Fed-batch fermentation, End-product inhibition, Anion exchange resin

## Abstract

Lactic acid bacteria are industrially important microorganisms recognized for fermentative ability mostly in their probiotic benefits as well as lactic acid production for various applications. Fermentation conditions such as concentration of initial glucose in the culture, concentration of lactic acid accumulated in the culture, types of pH control strategy, types of aeration mode and different agitation speed had influenced the cultivation performance of batch fermentation of *Pediococcus acidilactici*. The maximum viable cell concentration obtained in constant fed-batch fermentation at a feeding rate of 0.015 L/h was 6.1 times higher with 1.6 times reduction in lactic acid accumulation compared to batch fermentation. Anion exchange resin, IRA 67 was found to have the highest selectivity towards lactic acid compared to other components studied. Fed-batch fermentation of *P. acidilactici* coupled with lactic acid removal system using IRA 67 resin showed 55.5 and 9.1 times of improvement in maximum viable cell concentration compared to fermentation without resin for batch and fed-batch mode respectively. The improvement of the *P. acidilactici* growth in the constant fed-batch fermentation indicated the use of minimal and simple process control equipment is an effective approach for reducing by-product inhibition. Further improvement in the cultivation performance of *P. acidilactici* in fed-bath fermentation with in situ addition of anion-exchange resin significantly helped to enhance the growth of *P. acidilactici* by reducing the inhibitory effect of lactic acid and thus increasing probiotic production.

## Introduction

Lactic acid bacteria (LAB) have recently attracted greater attention for industrial use due to their microorganisms beneficial to human or animals and probiotic properties (Hwang et al. 2011). *Pediococcus acidilactici* is one of the probiotics which commonly found as one of the normal flora of alimentary tract, oral cavity gastro intestinal tract and has been widely used in the food industry (Halim et al. 2017). Foods that contain probiotic in an appropriate amount are beneficial to human for instance in enhancing immune system, antibiotics production and prevention of mucosal infection in children. Probiotic products are recommended to contain at least 10^7^ living microorganisms per mL or per g (Elsayed et al. 2014). In addition, foods and tablets consist of LAB have been manufactured and consumed clinically. Therefore, it is necessary for a suitable commercial product to contain a high density of viable cells for efficient effects (Cui et al. 2016).

High cell density during fermentation is important in order to achieve targeted biomass, lactic acid and other metabolites productivity. Nonetheless, the conditions of the fermentation medium such as pH, temperature, substrates and by-products are the main influences on the microbial growth and product formation (Boon et al. 2007). Generally, conducive biochemical and biophysical conditions are important for bacteria to grow and express normal metabolic activities. Biophysical conditions such as temperature, pH, redox potential, water activity and the presence of inhibitory compounds gave a broad range of variations among LAB strains. A suitable biochemical condition for LAB to grow can be obtained from nutrients provided by the culture media (Hayek and Ibrahim 2013).

Batch, fed-batch and continuous are three fermentation modes commonly used for biomass production in microbial fermentation. Among these, batch fermentation is identified to be the most frequently used for fermentation process due to the simplicity of the process where no substrates components are required to be added during the fermentation process except neutralizing agents for pH controlling (Abdel-Rahman et al. 2013). The closed system of batch fermentation minimize the risk of contamination and in turn produce a high lactic acid concentration as compared to other fermentation modes (Hofvendahl and Hahn-Hägerdal 2000). However, batch fermentation has the drawbacks of producing low cell concentrations mainly due to the limited nutrient levels (Abdel-Rahman et al. 2013) and the LAB culture often suffers from substrate and product inhibitions in which contributes to the low productivity (Hujanen et al. 2001).

Fed-batch fermentation is normally used in order to avoid substrate-level inhibition in microbial fermentation (Bai et al. 2003). In comparison, batch and fed-batch fermentations may produce higher lactic acid concentrations than a continuous fermentation. Often, this is due to the complete consumption of substrates available in the batch and fed-batch fermentations, whereas there are always remaining residual concentrations of substrates in the continuous fermentation (Hofvendahl and Hahn-Hägerdal 2000; Abdel-Rahman et al. 2013). In-situ removal of lactic acid from fermentation broth is another interesting strategy to reduce lactic acid product inhibition in the fermentation of LAB (Cui et al. 2016; Garret et al. 2015; Jianlong et al. 1994). Nonetheless, most of the studies on the extractive fermentation of lactic acid using anion exchange resin are focusing on the improvement of lactic acid production instead of cell biomass (Boonmee et al. 2016). Limited literature is therefore available on the combination of both lactic acid extractive fermentation using anion exchange resin and fed-batch fermentation aiming at the improvement of LAB biomass.

The purpose of this study was to investigate the effects of fermentation conditions on the growth of batch fermentation of *P. acidilactici* and to improve its cultivation performance through the application of constant fed-batch fermentation in 2 L stirred tank bioreactor. Fermentation conditions such as concentration of initial glucose in the culture, concentration of lactic acid accumulated in the culture, types of pH control strategy, types of aeration mode and different agitation speed were studied on batch fermentation of *P. acidilactici*. Whilst for the constant fed-batch fermentation, different feeding rates were used to study the effects of feeding rate on viable cells production and lactic acid accumulated from cultivation of *P. acidilactici.* Lactic acid extractive fermentation using anion exchange resin, IRA 67 was conducted on fed-batch fermentation of *P. acidilactici* to further improve its growth. The results of this study may provide several innovative strategies for improvement of biomass production in LAB fermentation.

## Materials and methods

### Microorganism, culture maintenance and inoculum preparation

*Pediococcus acidilactici* DSM 20238 used in this study was obtained from Bioprocessing and Biomanufacturing Research Center, Universiti Putra Malaysia which was initially purchased from DSMZ-German Collection of Microorganisms and Cell Cultures. This strain was used throughout the study due to its capability to produce lactic acid as a sole product. De Man, Rogosa and Sharpe (MRS) medium was used for growing of *P. acidilactici* strain and for maintenance of the culture. The MRS medium was purchased from Merck Millipore, Germany. The compositions of MRS medium in deionized water (g/L) were: peptone from casein 10.0, meat extract 8.0, yeast extract 4.0, d(+) glucose 20.0, di-potassium hydrogen phosphate 2.0, Tween 80 1.0, di-ammonium hydrogen citrate 2.0, sodium acetate 5.0, magnesium sulfate 0.2, and manganese sulfate 0.04. The strain was stored at − 20 °C in 15% (v/v) glycerol. The strain was inoculated into 50 mL MRS broth in 250 mL non-baffled Erlenmeyer flasks and incubated in an incubator shaker (Certomat^®^ BS-1 Braun, Germany) at 37 °C and agitated at 200 rpm for 24 h. The bacterial cells were harvested by centrifugation (Eppendorf Centrifuge 5810 R) at 10,000 rpm for 10 min. The bacterial pellets were resuspended in 15% (v/v) glycerol and stored at − 20 °C for maintenance. The 15% (v/v) glycerol was prepared by mixing with distilled water and autoclaving at 121 °C and 15 psi for 15 min for sterilization purpose. Inoculum was prepared by growing one loopful of *P. acidilactici* culture from MRS agar plate in 250 mL non-baffled Erlenmeyer flasks containing 50 mL MRS medium. The strain was incubated at 37 °C in an orbital shaker, which was agitated at 200 rpm for 10 h to be used as inoculum in fermentation process.

### Batch fermentation

For preliminary experiments, the fermentations were done in 500 mL non-baffled Erlenmeyer flasks containing 100 mL MRS medium with composition as previously described. Different glucose concentrations (0–20 g/L) were used in MRS medium to study the effect of glucose consumption and lactic acid accumulated in the culture on growth of *P. acidilactici*. Different lactic acid concentrations (5–15 g/L) were added into the culture during exponential phase of the bacterial growth to investigate the lactic acid inhibition towards the growth of *P. acidilactici*. Batch cultivation was carried out in a 2 L stirred tank bioreactor (BIOSTAT, B. Braun Biotech International, GmbH, Germany). The bioreactor vessel was made of borosilicate glass and the top-plate of the bioreactor was made of stainless steel. The bioreactor was equipped with a thermostat jacket system for temperature controlling within the bioreactor by means of an external double wall with a circulation pump. The bioreactor was connected with temperature, pH and dissolved oxygen monitoring and control system. pH of the culture was controlled by addition of sodium hydroxide (NaOH) or hydrochloric acid (HCl) accordingly. Alkali, acid and feed medium were added into the culture using peristaltic pumps. Antifoam was not added into the bioreactor since foaming was not a problem during fermentation. The bioreactor was equipped with a six blade Rushton turbine for mixing system. Batch cultivations were carried out in a 2 L stirred tank bioreactor containing 1 L of MRS medium. The culture in bioreactor was also inoculated with 10% (v/v) inoculum similar to the shake flask culture. The fermentations were carried out under facultative condition, agitated at 300 rpm, at 37 °C and the pH was not controlled but monitored throughout the fermentation, unless stated otherwise. The effect of fermentation conditions with and without pH control on the fermentation performance of *P. acidilactici* was studied by running the fermentation at both non-regulated pH and regulated pH value of 5.7 ± 0.2 (equivalent to the initial pH value of MRS broth). The initial pH values of these two conditions were adjusted by 1 M NaOH or HCl. Due to the lactic acid production during fermentation of *P. acidilactici*, the pH was maintained with 1 M NaOH during the fermentation for pH control condition. The effect of aeration on the fermentation performance of *P. acidilactici* was studied by running the fermentation with and without air supply. Due to the facultative characteristic of *P. acidilactici*, two different modes of aeration were selected to study the effect of aeration on the growth of *P. acidilactici*. For the anaerobic condition, the fermentation was done by sparging the medium with nitrogen gas at 0.1 vvm (0.15 L/min) until the dissolved oxygen level reached zero value prior to fermentation. Nitrogen gas was stopped once the culture was inoculated, and the fermentation was allowed to proceed without aeration. For the facultative condition, the fermentation was done by sparging the medium with oxygen gas at 0.1 vvm (0.15 L/min) until the dissolved oxygen level achieved 100% prior to fermentation. Oxygen gas was stopped once the culture was inoculated, then the fermentation proceeded without aeration. Finally, the effect of different mixing rate on the fermentation performance of *P. acidilactici*, was evaluated at three different mixing speeds of 200, 300 and 400 rpm.

#### Constant fed-batch fermentation

Constant fed-batch fermentations were conducted in a 2 L stirred tank bioreactor at three different feeding rates of 0.008, 0.015 and 0.03 L/h. Basically the constant fed-batch fermentations were conducted in two phases. In the initial phase, *P. acidilactici* was cultured in a batch fermentation mode with a working volume of 0.9 L until glucose was exhausted. Once the glucose was exhausted, which occurred approximately at 12 h of the initial batch fermentation, the second phase of the fermentation was started with fed-batch mode. During the second phase, the fed-batch was initiated with 100 mL of concentrated glucose (10 g/L) was being added continuously into the bioreactor at a constant feeding rate to make up the total volume of 1 L.

#### Anion-exchange resin selectivity towards lactic acid

A weak base anion-exchange resin (Amberlite IRA 67, Cat. No. 476633) was used in this study for in situ removal of lactic acid from the culture. The resin was purchased from Sigma, Germany and was sterilized using ultraviolet radiation before used in the experiments. The tubes were centrifuged at 10,000 rpm for 10 min to separate resins from the solution to be used for determination of remaining lactic acid in the solution. Resin can be reused by washing the resin with distilled water followed by regenerating with 4% NaOH to elute the lactic acid from the resin at an ambient temperature. The theory of the Langmuir isotherm model assumes adsorption only occurs at specific homogeneous adsorbent sites and no further adsorption can take place at the same site once a lactic acid molecule adsorbs on the site (Gao et al. 2010). The specific uptake capacity of lactic acid (q) for the Langmuir isotherm model was determined by using Eq. 1:

1 $$ q = \frac{(Ci - Ceq)}{X} $$

From Eq. 1, q (g of lactic acid/g of biosorbent) is the specific uptake capacity of lactic acid, C_i_ (g/L) is the initial concentration of lactic acid, C_eq_ (g/L) is the equilibrium concentration of lactic acid and X (g/L) is the concentration of biosorbent in solution. Sorption equilibrium studies for a single component present in MRS media (glucose and sodium acetate) and organic acid other than lactic acid (acetic acid) were conducted to study the selectivity of IRA 67 anion-exchange resin towards these components as compared to lactic acid. IRA 67 resin (10 g/L) were added into 15 mL falcon tubes with each separately contained 10 mL glucose, sodium acetate and acetic acid with initial concentration of 5 g/L. The tubes were agitated at 200 rpm on a shaker for 24 h until the sorption reached equilibrium. The adsorption capacity of IRA 67 resin for glucose, sodium acetate and acetic acid was calculated using Eq. 1 by determining the remaining of these components in the solution. The experiments were done in triplicate.

#### Constant fed-batch fermentation coupled with lactic acid extractive fermentation using anion-exchange resin

The cultivation performance of *P. acidilactici* in the constant fed-batch fermentation coupled with extractive fermentation using the condition of dispersed anion-exchange resin were conducted at feeding rate of 0.015 L/h. The sterilized an anion-exchange resin was aseptically added (10 g/L) into the culture during inoculation. Fermentation culture was centrifuged at 10,000 rpm for 10 min to separate resins from the culture to be used for further application. Resin can be reused by washing and regenerating as mentioned in the previous section.

#### Analytical methods

Throughout the fermentations, 5 mL of culture samples were withdrawn at time intervals for analysis. Cell growth was determined using colony forming unit (CFU) (Ming et al. 2016). Samples were serially diluted (10^3^–10^10^) with 0.9% (w/v) sterile saline water (NaCI) and plated onto MRS agar plate. Samples were incubated for 24 h at 37 °C and were analyzed for CFU/mL by calculating the total number of colonies on a plate and multiplied by the dilution involved. The supernatant was separated from the broth by centrifugation at 10,000 rpm for 10 min to be used for glucose and lactic acid determination. The concentrations of glucose and lactic acid were determined by using reverse-phase high performance liquid chromatography (RP-HPLC) (Waters 2695, Separations Module and Waters 2410, Refractive Index Detector). The RP-HPLC analysis was done using a shodex SH-1011 column (7 μm, 8 mm × 300 mm) connected with shodex SH-G guard column (7 μm, 6 × 50 mm). The mobile phase solvent used was 5 mM sulphuric acid, the temperature was maintained at 60 °C and the flow rate was 1.0 mL/min. Empower software was used for data processing.

#### Statistical analysis

The experiments data were statistically analysed by using MS Office Excel 2010 and SPSS software Version 21. The data represents averages of at least three replicates. The results were represented as mean value ± standard deviation. Unpaired T test and one way analysis of variance (One way ANOVA) were used to determine the significance among treatment means. Significance was declared at P < 0.05.

## Results

### Effect of glucose concentration on growth of *P. acidilactici* and lactic acid accumulation

**Fig. 1:**
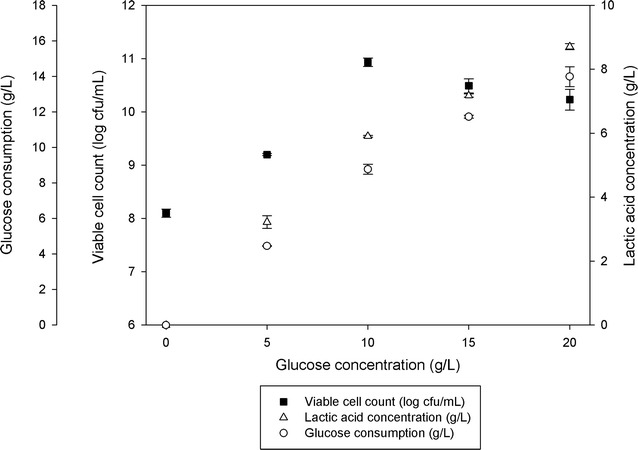
Effect of glucose concentration on growth of *P. acidilactici* and lactic acid accumulation. The fermentation was conducted in 500 mL shake flask, at 200 rpm. The data are the average of triplicate experiments. The error bars represent the standard deviations about the mean (n = 3)

Varying the initial glucose concentration from 0 to 20 g/L in batch fermentation was found to affect the growth of *P. acidilactici* (Fig. 1). Increased in initial glucose concentration from 0 to 10 g/L increased viable cell concentration of *P. acidilactici* (from 9.4 × 10^8^ to 1.4 × 10^10^ cfu/mL) by about 14.9 times whilst at glucose concentration of above 10–20 g/L viable cell concentration of *P. acidilactici* was reduced by about 1.4 times (from 1.4 × 10^10^ to 1.0 × 10^10^ cfu/mL). Viable cell concentration obtained and lactic acid accumulated at the end of the fermentation were significantly different (P < 0.05) at different initial glucose concentrations studied. Although the growth of *P. acidilactici* was slightly reduced as glucose concentration was increased from above 10 to 20 g/L, however, lactic acid accumulation and glucose consumption were still increased from 5.91 to 8.71 g/L and 8.77 to 13.59 g/L, respectively.

### Effect of lactic acid concentration on growth inhibition of *P. acidilactici*

Different lactic acid concentrations (0, 5, 10 and 15 g/L) were added to the culture at exponential phase of growth (8 h) to study the effect of lactic acid on viability of *P. acidilactici* after exponential phase. Lactic acid was added at exponential phase instead of at the beginning of the fermentation due to the failure of cells to grow in highly acidic condition during initial or lag phase (data not shown). As tabulated in Table 1, final viable cell concentration obtained at the end of the fermentation was significantly different (P < 0.05) at different lactic acid concentrations added. Final viable cell concentration of *P. acidilactici* was decreased proportionally with increased of lactic acid concentration. The final viable cell concentration reduced from 9.1 × 10^9^ to 8.2 × 10^7^ cfu/mL when 5 g/L of lactic acid was added. The final viable cell concentration was then further reduced to 8.4 × 10^4^ cfu/mL when 10 g/L of lactic acid was added and no cells were survived when 15 g/L of lactic acid was added to the culture.

**Table 1:** Viability of *P. acidilactici* in 500 mL shake flask at different lactic acid concentrations

Lactic acid added (g/L)	Lactic acid accumulated (g/L)^e^	Final viable cell concentration (cfu/mL)
0	6.47 ± 0.23^d^	9.1 × 10^9^ ± 0.12^a^
5	11.33 ± 0.20^c^	8.2 × 10^7^ ± 0.35^b^
10	16.52 ± 0.18^b^	8.4 × 10^4^ ± 0.27^c^
15	21.89 ± 0.14^a^	0.0

The results presented are the average of triplicate experiments and are expressed as mean ± standard deviation

^a,b,c,d^Mean values in the same row with different superscripts are significantly different from each other (P < 0.05)

^e^Lactic acid accumulated is the total of lactic acid added to the culture and lactic acid produced from fermentation

### Effect of pH control strategy on growth of *P. acidilactici* in batch cultivation using 2 L stirred tank bioreactor

**Fig. 2:**
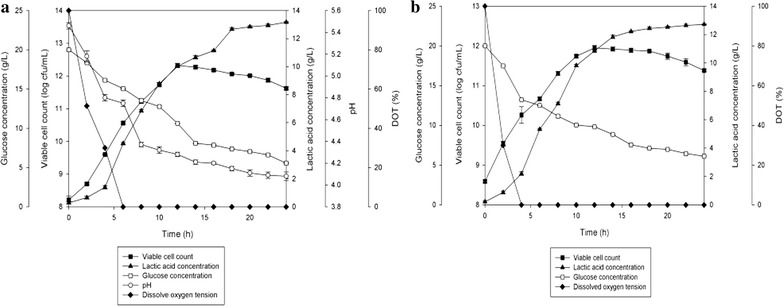
The time course of batch fermentation of *P. acidilactici* in 2 L stirred tank bioreactor **a** without pH control, **b** with pH control at pH 5.7. The fermentation was conducted at 300 rpm. The error bars represent the standard deviations about the mean (n = 3)

In order to study the inhibitory effect of pH reduction due to lactic acid production from fermentation of *P. acidilactici* on its growth, batch fermentations of *P. acidilactici* with and without pH control conditions were conducted. The time course of the fermentation is shown in Fig. 2 and the performance of the cultivation is summarized in Table 2. In the culture without pH control condition, the maximum viable cell concentration (1.5 × 10^12^ cfu/mL) was 8.3 times higher than the culture with pH control condition (1.8 × 10^11^ cfu/mL). Furthermore, viable cell yield and viable cell productivity obtained in the culture without pH control were also 8.9 times and 8.0 times respectively, higher than that obtained in the culture with pH control. Maximum viable cell concentration, viable cell yield and viable cell productivity obtained were significantly different (*P* < 0.05) at both with and without pH control conditions. These results hence proved that the addition of NaOH to control the pH was not effective in reducing end-product inhibition in fermentation of *P. acidilactici*. There were significant differences (*P* < 0.05) in lactic acid produced and lactic acid productivity for both types of cultures. Lactic acid produced in fermentation without pH control condition (13.17 g/L) was 1.0 time higher compared to the fermentation with pH control condition (12.72 g/L) and lactic acid productivity in fermentation without pH control condition (0.54 g/L h) was 1.03 times higher compared to the fermentation with pH control condition (0.52 g/L h). Lactic acid accumulated was slightly lower in fermentation with NaOH addition due to lactic acid being neutralized. However, lactic acid yield was similar for both types of cultures (0.90 g/g_Glucose_), showing no significant difference (*P* < 0.05) in lactic acid yield for both with and without pH control conditions.

**Table 2:** Effect of culture pH on growth of *P. acidilactici* in batch fermentation using 2 L stirred tank bioreactor

Kinetic parameter	No pH control	pH 5.7
Maximum viable cell concentration (cfu/mL)	1.5 × 10^12^ ± 0.17^a^	1.8 × 10^11^ ± 0.24^b^
Time to reach maximum viable cell concentration (h)	12^a^	12^a^
Viable cell yield (cfu/g_Glucose_)	1.6 × 10^14^ ± 0.15^a^	1.8 × 10^13^ ± 0.12^b^
Viable cell productivity (cfu/mL h)	1.2 × 10^11^ ± 0.12^a^	1.5 × 10^10^ ± 0.18^b^
Maximum lactic acid concentration (g/L)	13.17 ± 0.08^a^	12.72 ± 0.1^b^
Lactic acid yield (g/g_Glucose_)	0.90 ± 0.05^a^	0.90 ± 0.06^a^
Lactic acid productivity (g/L h)	0.54 ± 0.05^a^	0.52 ± 0.08^b^

The results presented are the average of triplicate experiments and are expressed as mean ± standard deviation

^a,b^Mean values in the same row with different superscripts are significantly different from each other (P < 0.05)

### Effect of aeration on growth of *P. acidilactici* in batch cultivation using 2 L stirred tank bioreactor

**Fig. 3:**
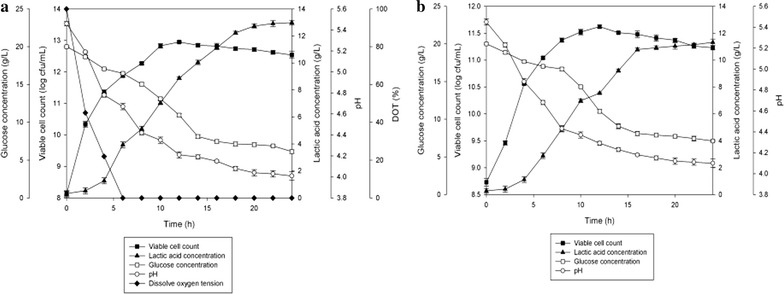
The time course of batch fermentation of *P. acidilactici* in 2 L stirred tank bioreactor at condition of **a** facultative, **b** anaerobic. The fermentation was conducted at 300 rpm. The error bars represent the standard deviations about the mean (n = 3)

For facultative microorganisms such as *P. acidilactici*, facultative condition was shown to improve the cultivation performance of *P. acidilactici* compared to anaerobic condition (Fig. 3). As shown in Table 3, maximum viable cell concentration, viable cell yield and viable cell productivity obtained were slightly higher in facultative condition (1.7 × 10^12^ cfu/mL, 1.9 × 10^14^ cfu/g_Glucose_ and 1.4 × 10^11^ cfu/mL h, respectively) compared to anaerobic condition (1.2 × 10^11^ cfu/mL, 1.3 × 10^13^ cfu/g_Glucose_ and 9.9 × 10^9^ cfu/mL h, respectively). Maximum viable cell concentration, viable cell yield and productivity obtained were significantly different (*P* < 0.05) at both facultative and anaerobic conditions. Similar to the results of maximum viable cell concentration, yield and productivity, the same trend was obtained for lactic acid production, yield and productivity, where the results were slightly higher in facultative condition (12.98 g/L, 0.91 g/g_Glucose_ and 0.53 g/L h respectively) compared to anaerobic condition (11.32 g/L, 0.86 g/g_Glucose_ and 0.46 g/L h respectively). There were significant differences (*P* < 0.05) in lactic acid production, yield and productivity obtained at both facultative and anaerobic fermentations.

**Table 3:** Effect of aeration on growth of *P. acidilactici* in batch fermentation using 2 L stirred tank bioreactor

Kinetic parameter	Facultative	Anaerobic
Maximum viable cell concentration (cfu/mL)	1.7 × 10^12^ ± 0.13^a^	1.2 × 10^11^ ± 0.21^b^
Time to reach maximum viable cell concentration (h)	12^a^	12^a^
Viable cell yield (cfu/g_Glucose_)	1.9 × 10^14^ ± 0.12^a^	1.3 × 10^13^ ± 0.17^b^
Viable cell productivity (cfu/mL h)	1.4 × 10^11^ ± 0.1^a^	9.9 × 10^9^ ± 0.13^b^
Maximum lactic acid concentration (g/L)	12.98 ± 0.21^a^	11.32 ± 0.23^b^
Lactic acid yield (g/g_Glucose_)	0.91 ± 0.18^a^	0.86 ± 0.21^b^
Lactic acid productivity (g/L h)	0.53 ± 0.14^a^	0.46 ± 0.17^b^

The results presented are the average of triplicate experiments and are expressed as mean ± standard deviation

^a,b^Mean values in the same row with different superscripts are significantly different from each other (P < 0.05)

### Effect of agitation speed on growth of *P. acidilactici* in batch cultivation using 2 L stirred tank bioreactor

**Fig. 4:**
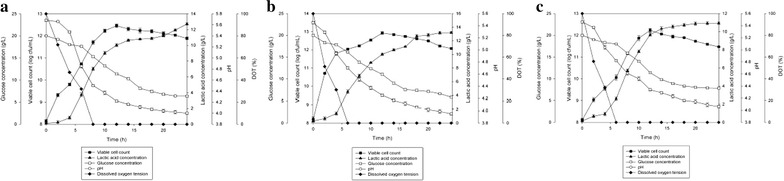
The time course of batch fermentation of *P. acidilactici* in 2 L stirred tank bioreactor at agitation speed of **a** 200 rpm, **b** 300 rpm and **c** 400 rpm. The error bars represent the standard deviations about the mean (n = 3)

The growth profiles for batch fermentation of *P. acidilactici* in 2 L stirred tank bioreactor at different agitation speeds are shown in Fig. 4. Increased in agitation speed from 200 to 300 rpm showed improvement in maximum viable cell concentration, viable cell yield, viable cell productivity, lactic acid production, lactic acid yield and lactic acid productivity (Table 4). However, as the agitation speed was increased from 300 to 400 rpm, the cultivation performance of *P. acidilactici* was declined. Results from this study demonstrated that agitation speed had an influence on growth of *P. acidilactici.* Of all the three agitation speeds studied, 300 rpm showed the highest maximum viable cell concentration (1.8 × 10^12^ cfu/mL) with improvement of 1.5 times and 1.8 times compared to 200 (1.2 × 10^12^ cfu/mL) and 400 rpm (1.0 × 10^12^ cfu/mL), respectively. This shows that agitation speed of 300 rpm provides suitable degree of mixing for growth of *P. acidilactici* compared to other agitation speeds.

Glucose consumption by *P. acidilactici* was found to be increased from 200 (68.2%) to 300 rpm (71%) and then reduced at 400 rpm (63%). The highest viable cell yield was obtained at 300 rpm (2.0 × 10^14^ cfu/g_Glucose_), followed by 200 (1.4 × 10^14^ cfu/g_Glucose_) and 400 rpm (1.2 × 10^14^ cfu/g_Glucose_). This trend was similar to the viable cell productivity where 300 rpm showed the highest viable cell productivity (1.5 × 10^11^ cfu/mL h) compared to 200 (9.9 × 10^10^ cfu/mL h) and 400 rpm (8.3 × 10^10^ cfu/mL h). Maximum viable cell concentrations, viable cell yield and productivity obtained were significantly different (*P* < 0.05) at all of the three agitation speeds studied. The highest total lactic acid produced from the fermentation of *P. acidilactici* was found at 300 rpm (13.21 g/L) followed by 200 (12.71 g/L) and 400 rpm (11.77 g/L), showing significant difference (*P* < 0.05) in lactic acid production for all of the agitation speeds studied. Nonetheless, lactic acid yield obtained were almost similar at all of the three agitation speeds, showing no significant difference (*P* < 0.05) for all of the cultures. As for the lactic acid productivity, cultures at 200 and 300 rpm showed almost similar results with no significant difference (*P* < 0.05), whilst culture at 400 rpm attained the lowest lactic acid productivity.

**Table 4:** Effect of agitation speed on growth of *P. acidilactici* in batch fermentation using 2 L stirred tank bioreactor

Kinetic parameter	Agitation speed (rpm)		
	200	300	400
Maximum viable cell concentration (cfu/mL)	1.2 × 10^12^ ± 0.17^b^	1.8 × 10^12^ ± 0.12^a^	1.0 × 10^12^ ± 0.23^c^
Time to reach maximum viable cell concentration (h)	12^a^	12^a^	12^a^
Viable cell yield (cfu/g_Glucose_)	1.4 × 10^14^ ± 0.18^b^	2.0 × 10^14^ ± 0.1^a^	1.2 × 10^14^ ± 0.17^c^
Viable cell productivity (cfu/mL h)	9.9 × 10^10^ ± 0.16^b^	1.5 × 10^11^ ± 0.15^a^	8.3 × 10^10^ ± 0.12^c^
Maximum lactic acid concentration (g/L)	12.71 ± 0.16^b^	13.21 ± 0.14^a^	11.77 ± 0.23^c^
Lactic acid yield (g/g_Glucose)_	0.91 ± 0.07^a^	0.92 ± 0.08^a^	0.91 ± 0.12^a^
Lactic acid productivity (g/L h)	0.53 ± 0.08^a^	0.54 ± 0.08^a^	0.48 ± 0.1^b^

The results presented are the average of triplicate experiments and are expressed as mean ± standard deviation

^a,b,c^Mean values in the same row with different superscripts are significantly different from each other (P < 0.05)

### Effect of feed rate in constant fed-batch fermentation on growth of *P. acidilactici* using 2 L stirred tank bioreactor

**Fig. 5:**
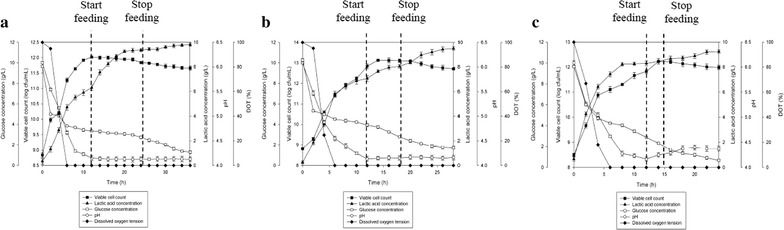
The time course of constant fed-batch fermentation of *P. acidilactici* in 2 L stirred tank bioreactor at feeding rate of **a** 0.008 L/h, **b** 0.015 L/h and **c** 0.03 L/h. The error bars represent the standard deviations about the mean (n = 3)

Figure 5 depicted the time course of constant fed-batch fermentation of *P. acidilactici* in 2 L stirred tank bioreactor with different feeding rate of limiting substrate. As tabulated in Table 5, increased feeding rate from 0.008 to 0.015 L/h, showed 5.8 times improvement in maximum viable cell concentration from 1.9 × 10^12^ to 1.1 × 10^13^ cfu/mL. However, as the feeding rate was further increased from 0.015 to 0.03 L/h, maximum viable cell concentration obtained was reduced 6.9 times equivalent to 1.6 × 10^12^ cfu/mL. Maximum viable cell concentration obtained was significantly different (*P* < 0.05) at all of the three feeding rate.

The highest viable cell yield was obtained at 0.015 L/h (1.2 × 10^15^ cfu/g_Glucose_), followed by 0.008 (2.1 × 10^14^ cfu/g_Glucose_) and 0.03 L/h (1.8 × 10^14^ cfu/g_Glucose_). Whilst, the highest viable cell productivity was obtained at 0.015 L/h (7.9 × 10^11^ cfu/mL h) followed by 0.03 (1.1 × 10^11^ cfu/mL h) and 0.008 L/h (1.1 × 10^10^ cfu/mL h). Viable cell yield and viable cell productivity obtained were significantly different (*P* < 0.05) at all of the three feeding rate studied.

As for the lactic acid production, the highest lactic acid produced from constant fed-batch fermentation of *P. acidilactici* was found at feeding rate 0.008 L/h (9.81 g/L) followed by 0.03 (9.25 g/L) and 0.015 L/h (8.12 g/L). Nevertheless, lactic acid yield at feeding rate of 0.008 and 0.03 L/h were almost similar (1.09 and 1.05 g/g_Glucose_ respectively) with no significant difference (*P* < 0.05), whilst lactic acid yield at feeding rate of 0.015 L/h was slightly lower (0.89 g/g_Glucose_) showing significant difference (*P* < 0.05) for lactic acid yield at feeding rate of 0.015 L/h. As for the lactic acid productivity, culture with feeding rate of 0.008 and 0.015 L/h showed almost similar results (0.26 and 0.28 g/L h) with no significant difference (*P* < 0.05) at both feeding rate, whilst culture at 0.03 L/h showed the highest lactic acid productivity. Improvement of 6.1 times in maximum viable cell concentration and reduction of 1.6 times in lactic acid production were achieved in constant fed-batch fermentation as compared to batch fermentation. There were significant differences (*P* < 0.05) in maximum viable cell concentration, viable cell yield, viable cell productivity, lactic acid production, yield and productivity obtained for both batch and fed-batch fermentations.

**Table 5:** Effect of feeding rate on growth of *P. acidilactici* in constant fed-batch fermentation using 2 L stirred tank bioreactor

Kinetic parameter	Feeding rate (L/h)		
	0.008	0.015	0.03
Maximum viable cell concentration (cfu/mL)	1.9 × 10^12^ ± 0.21^b^	1.1 × 10^13^ ± 0.14^a^	1.6 × 10^12^ ± 0.12^c^
Time to reach maximum viable cell concentration (h)	12^b^	14^a^	14^a^
Viable cell yield (cfu/g_Glucose_)	2.1 × 10^14^ ± 0.16^b^	1.2 × 10^15^ ± 0.11^a^	1.8 × 10^14^ ± 0.13^c^
Viable cell productivity (cfu/mL h)	1.1 × 10^10^ ± 0.18^c^	7.9 × 10^11^ ± 0.12^a^	1.1 × 10^11^ ± 0.17^b^
Maximum lactic acid concentration (g/L)	9.81 ± 0.12^a^	8.12 ± 0.11^c^	9.25 ± 0.14^b^
Lactic acid yield (g/g_Glucose_)	1.09 ± 0.08^a^	0.89 ± 0.06^b^	1.05 ± 0.06^a^
Lactic acid productivity (g/L h)	0.26 ± 0.11^b^	0.28 ± 0.08^b^	0.36 ± 0.07^a^

The results presented are the average of triplicate experiments and are expressed as mean ± standard deviation

^a,b,c^Mean values in the same row with different superscripts are significantly different from each other (P < 0.05)

### Anion-exchange resin selectivity towards lactic acid

Table 6 shows the sorption study of lactic acid, acetic acid, sodium acetate and glucose on IRA 67 resin when these components were present separately and in a pure form. From the sorption study and the amount of components adsorbed as calculated from Eq. 1, it was found that the adsorption of lactic acid on Amberlite IRA 67 resin was significantly different (*P* < 0.05) from the adsorption of acetic acid, sodium acetate and glucose. It was also found that the highest adsorbed component by IRA 67 resin was lactic acid, followed by acetic acid, sodium acetate and glucose.

**Table 6:** Selectivity of Amberlite IRA 67 resin (10 g/L) towards lactic acid, acetic acid, glucose and sodium acetate

Component	Adsorption		
	Initial concentration (g/L)	Equilibrium concentration (g/L)	Amount adsorbed (g/g)
Lactic acid	5	1.02 ± 0.09	0.40 ± 0.10^a^
Acetic acid	5	1.84 ± 0.13	0.32 ± 0.11^b^
Sodium acetate	5	2.96 ± 0.08	0.20 ± 0.09^c^
Glucose	5	3.23 ± 0.12	0.18 ± 0.09^c^

The results of equilibrium concentration and amount adsorbed are the average of triplicate experiments

Statistically significant coefficient (P < 0.05) are expressed as mean ± SE

^a,b,c^Mean values with the different letters are significantly different

### Constant fed-batch fermentation coupled with lactic acid extractive fermentation using anion-exchange resin

**Fig. 6:**
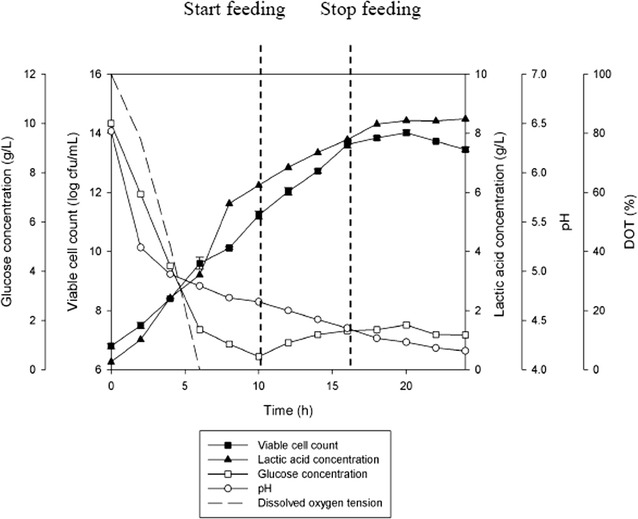
The time course of constant fed-batch fermentation of *P. acidilactici* in 2 L stirred tank bioreactor with in situ addition of 10 g/L IRA 67 resin. The error bar represents the standard deviation about the mean (n = 3)

Figure 6 shows the results for fed-batch fermentation coupled with extractive fermentation using IRA 67 anion-exchange resin that was conducted at the best fermentation conditions as observed from the previous sections (glucose concentration of not more than 10 g/L, fermentation without pH control, fermentation with facultative condition, agitation speed of 300 rpm and feeding rate of 0.015 L/h). As tabulated in Table 7, the growth of *P. acidilactici* with resin addition for fed-batch fermentation was found to be improved by 9.1 times (1.0 × 10^14^ cfu/mL) compared to the conventional fed-batch fermentation without resin addition (1.1 × 10^13^ cfu/mL). However, the time taken for the culture to reach maximum viable cell concentration was 6 h longer in the fed-batch fermentation with resin compared to without resin. Viable cell yield and viable cell productivity obtained in the culture with resin were 8.5 times and 8.6 times higher, respectively, than that obtained in the culture without resin. There were significant differences (*P* < 0.05) in maximum viable cell concentration, yield and productivity for both fed-batch fermentations with and without resin addition. Lactic acid accumulated in the culture was lower in the fed-batch fermentation with resin addition (8.78 g/L) compared to fed-batch fermentation without resin addition (9.62 g/L). However, the total lactic acid produced was higher in fed-batch fermentation with resin (12.24 g/L) compared to fed-batch fermentation without resin (9.62 g/L). On the other hand, lactic acid yield for fed-batch fermentation with resin was lower (0.72 g/g_Glucose_) than fed-batch fermentation without resin (0.84 g/g_Glucose_) and lactic acid productivity was higher in the culture with resin (0.43 g/L h) compared to the culture without resin (0.33 g/L h). There were significant differences (*P* < 0.05) in lactic acid production, yield and productivity obtained for both fed-batch fermentations with and without resin addition.

**Table 7:** Effect of resin addition on growth of *P. acidilactici* in fed-batch fermentation using 2 L stirred tank bioreactor

Kinetic parameter	Fed-batch without resin	Fed-batch with resin
Maximum viable cell concentration (cfu/mL)	1.1 × 10^13^ ± 0.14^b^	1.0 × 10^14^ ± 0.07^a^
Time to reach maximum viable cell concentration (h)	14^b^	20^a^
Viable cell yield (cfu/g_Glucose_)	1.2 × 10^15^ ± 0.11^b^	7.2 × 10^16^ ± 0.12^a^
Viable cell productivity (cfu/mL h)	7.9 × 10^11^ ± 0.12^b^	5.1 × 10^12^ ± 0.08^a^
Lactic acid accumulated (g/L)	8.12 ± 0.11^b^	8.78 ± 0.06^a^
Lactic acid produced (g/L)	8.12 ± 0.11^b^	12.24 ± 0.08^a^
Lactic acid yield (g/g_Glucose_)	0.89 ± 0.06^a^	0.72 ± 0.06^b^
Lactic acid productivity (g/L h)	0.28 ± 0.08^b^	0.43 ± 0.04^a^

The results presented are the average of triplicate experiments

Statistically significant coefficient (*P* < 0.05) are expressed as mean ± SE

^a,b^Mean values with the different letters are significantly different

## Discussion

Reduced viable cell concentration with increased glucose concentration was due to high lactic acid accumulation in the culture since *P. acidilactici* undergo homofermentation. Generally, lactic acid conversion rate from sugar surpass 80% of the theoretical yield for homofermentative bacteria (Liu et al. 2013) and fermentation in *lactobacilli* tends to redirect its carbon flux from cell built-up to lactic acid production (Ming et al. 2016).

The results from the present study (Table 1) are similar to the findings of Monteagudo et al. (1997), who reported that there was an inhibition on bacterial growth by lactic acid when the lactic acid was rapidly being produced after the exponential phase of the growth. Reduced in final viable cell concentration of *P. acidilactici* was associated with high lactic acid concentration in the culture. This was due to the acidification of cytoplasm and failure of proton motive forces (Wee et al. 2006). The acidification of cytoplasm is causes by the undissociated lactic acid that passes through the bacterial membrane and dissociates inside the cell. The undissociated lactic acid is soluble within the cytoplasmic membrane whilst the dissociated lactate is insoluble. Eventually, this affects the transmembrane pH gradient and reduces the amount of energy that may be used for cell growth. Since lactic acid produced from fermentation causes acidification of medium and cytoplasm which inhibits the bacterial growth, therefore lactic acid produced in the culture must be either neutralized or removed as it is formed in order to maintain the pH within the optimal range (pH 5–7) (Nomura et al. 1987; Roberto et al. 2007).

The maximum viable cell concentration of *P. acidilactici* reduced when NaOH was added because the additional ions from NaOH increased the osmotic pressure of the medium, causing reduced cell growth (Cui et al. 2016). In addition, the accumulated lactic acid was not completely neutralized by NaOH because weak organic acids such as lactic acid and acetic acid are less dissociated in solution at any pH values compared to strong acids (Lund et al. 2014). Therefore, only part of the lactic acid was dissociated into lactate ions and the undissociated lactic acid which is membrane soluble will enter the cytoplasm via simple diffusion and dissociate inside the cell causing acidification of cytoplasm (Wee et al. 2006). In addition, the dissociated lactate ions can combine with external protons present and enter the cytoplasm in undissociated lactic acid form (Lund et al. 2014). The diffusion of lactic acid into the cytoplasm causes lactic acid to be rapidly dissociated releasing protons and anions within the cytoplasm (Broadbent et al. 2010). If the acidification of cytoplasm exceeds the buffering capacity of cytoplasmic and capabilities of efflux systems, the internal pH of the cell will drop and eventually causing failure in maintaining pH gradient and thus damage the cellular functions.

 Facultative condition was shown to improve the cultivation performance of *P. acidilactici* compared to anaerobic condition, and the result is in agreement with the observation reported by Smetankova et al. (2012) where improved growth in the presence of oxygen was observed in cultivation of three wild strains of *Lactobacillus plantarum* compared to anaerobic condition. This was due to the lactate dehydrogenase (LDH) being used in conversion of pyruvate to lactate under anaerobic condition, reducing regeneration of coenzyme nicotinamide adenine dinucleotide (NAD^+^) by LDH, where NAD^+^ is needed in metabolism of sugar. Whereas, under facultative or aerobic conditions, instead of LDH, nicotinamide adenine dinucleotide (NADH) oxidases and NADH peroxidases are being used for regeneration of NAD^+^. This causing the microorganism to redirect the flux, producing more adenosine triphosphate (ATP) and thus, providing more energy for growth (Condon 1987). Oxygen is an important factor for survival and mortality of aerobic microorganism but not to the facultative anaerobe (Duwat et al. 2001). A few studies have revealed that under fermentation condition, oxygen was shown to contribute toxic effects on *Lactococcus lactis* by inhibiting its growth and survival (Duwat et al. 1995; Condon 1987). Prolonged aeration of lactococcal cultures can cause DNA alteration and cell death. Formation of hydroxyl radicals and hydrogen peroxide may be the cause of the oxygen toxicity (Anders et al. 1970). Therefore, in this study, oxygen was not supply throughout the fermentation, instead, oxygen was only supply at the beginning of the fermentation before inoculation until dissolved oxygen level reached 100% and then the aeration was stopped to create facultative condition for the fermentation to progress facultatively. Furthermore, *P. acidilactici* is categorized as facultative anaerobe (Papagianni and Anastasiadou 2009), hence, the influence of DOT or oxygen transfer rate contributed by agitation speed on growth of *P. acidilactici* may not be crucial.

As illustrated in Fig. 5b, it can be observed that glucose concentration was maintained at a very low level (< 1.0 g/L) during the fed-batch fermentation with constant feeding of glucose at 0.015 L/h feeding rate. It can be concluded that the glucose consumption rate and the propagation of cells have achieved a quasi-steady state due to cell growth rate (dX/dt) = substrate consumption rate (dS/dt) = 0 for 6 h. This situation happened when substrate added was utilized by the cells as it was added into the culture. When the glucose addition was stopped, the cells functioned as resting cells. However, lactic acid production was still continued until the culture reached death phase at approximately 22 h of fermentation. Although glucose concentration in the culture at feeding rate of 0.008 L/h was similar to the culture at 0.015 L/h, however, maximum viable cells concentration obtained at constant feeding rate of 0.008 L/h was significantly (*P* < 0.05) lower (Fig. 5a). This may be due to the slow feeding of glucose which restricted the growth or growth inhibition by lactic acid accumulated in the culture. These assumptions are supported by the declining in viable cell concentration as observed at approximately 20 h of fermentation although glucose was still being added into the culture. When high feeding rate (0.03 L/h) was used, maximum viable cell concentration obtained was the lowest compared to other two feeding rate studied. It is tempting to speculate that fast addition of glucose at 0.03 L/h feeding rate has contributed to cells dilution and thus reduced maximum viable cell concentration in the culture. This assumption is supported by the high glucose accumulation recorded as soon as the feeding rate was started and declined viable cell concentration observed at approximately 16 h of fermentation. This situation, in a way, indicated that glucose consumption by *P. acidilactici* was affected when glucose feeding rate exceeded both substrate consumption rate and cell growth rate, which in turn, inhibited the growth.

Based on the results obtained, application of fed-batch fermentation reduced the product inhibition caused by undissociated lactic acid accumulation in the culture. The improvement of cultivation performance of *P. acidilactici* in the constant fed-batch fermentation was achieved due to the suitable environment conditions created by this method, such as glucose concentration of below inhibitory level and low lactic acid production due to glucose metabolic flux towards cell growth instead of lactic acid production. Fed-batch fermentation has the advantage of reducing extended lag phase of low cell density in batch fermentation (Aguirre-Ezkauriatza et al. 2010). In addition, substrate exhaustion can also be assured in fed-batch fermentation. Often, the achievement of high biomass concentrations in fed-batch fermentation managed to partially overcome the strong inhibition effect of lactic acid. Results from this study showed that the application of fed-batch fermentation in cultivation of *P. acidilactici* has significantly reduced the accumulation of lactic acid in the culture with significant increased in the viable cell number. This finding is in agreement with the study conducted by Ming et al. (2016), who found that glucose metabolic flux towards cell growth was increased with a reduction in lactic acid production through fed-batch fermentation of *Lactobacillus salivarius* I 24. In past studies conducted, improvement of productivity and final yield of targeted product with shorter fermentation time in different fermentation media has been successfully achieved through the application of fed-batch fermentation (Lee et al. 2007). Approximately two times higher final cell concentration and beyond 100% improvement in cell growth rate can be obtained by the application of fed-batch fermentation compared to batch fermentation. Besides, the application of fed-batch fermentation has also been widely applied in improving growth and biomass production of LAB such as in the production of *Lactobacillus plantarum* LP02 biomass isolated from infant feces with potential cholesterol lowering ability by Hwang et al. (2011) and improvement of cell mass production of *Lactobacillus delbrueckii* sp. *bulgaricus* WICC-B-02, a newly isolated probiotic strain from mother’s milk by Elsayed et al. (2014).

Amberlite IRA 67 anion-exchange resin was selected as the candidate to explore fed-batch couple with extractive fermentation of lactic acid is mainly due to the capability of this resin to effectively adsorb lactic acid in lactic acid fermentation (Garret et al. 2015). Amberlite IRA 67 is based on a matrix of cross linked acrylic gel which is more hydrophilic than styrenic resins and their selectivity to most of organic acids is higher due to their matrix (Arup 1995). Although the presence of other components (acetic acid, sodium acetate and glucose) did interfere with the adsorption capacity of IRA 67 resin towards lactic acid, however the affinity of IRA 67 resin was higher towards lactic acid than the other components studied.

Fed-batch mode was found to be superior to the batch mode of fermentation for the growth of *P. acidilactici*. Hence, the fed-batch fermentation of *P. acidilactici* was further extended with the application of in situ lactic acid removal system. Lactic acid accumulated in the culture was lower in the fed-batch fermentation with resin addition compared to fed-batch fermentation without resin addition due to the adsorption of lactic acid by the resin. The results of this study clearly shown that the in situ addition of anion-exchange resin in the fed-batch fermentation significantly helped to enhance the growth of *P. acidilactici* by reducing the inhibitory effect of lactic acid.

## Abbreviations

ATP: adenosine triphosphate
CFU: colony forming unit
DOT: dissolved oxygen tension
HCI: hydrochloric acid
LAB: lactic acid bacteria
LDH: lactate dehydrogenase
MRS: De Man Rogosa and Sharpe
NAD^+^: nicotinamide adenine dinucleotide
NADH: nicotinamide adenine dinucleotide
NaOH: sodium hydroxide
RP-HPLC: reverse-phase high performance liquid chromatography
rpm: rotation per minute
v/v: volume per volume
vvm: volumetric air flow rate
w/v: weight/volume
